# Book Reviews

**Published:** 1877-03

**Authors:** 


### Book Reviews.

A Century of American Medicine. 1776—1876. By *Edward H. Clarke, M.D.*, late Professor of Materia Medica in Harvard University, etc; *Henry J. Bigelow, M.D.*, Professor of Surgery in Harvard University, etc; *Samuel D. Gross, M.D., LL.D., D.C.L., Oxon.*, Professor of Surgery in the Jefferson Medical College, Phila., etc; *T. Gaillard Thomas, M.D.*, Professor of Obstetrics, etc., in the College of Physicians and Surgeons, New York, etc.; and *J. S. Billings, M. D.*, Librarian to the National Medical Library, Washington, D. C. Philadelphia: Henry C. Lea. 1876.

The profession will welcome this addition to our literature.

As a record of American Medicine, during the last hundred years, it is of great value. But that is not its only merit. The eminent authors of the five papers which constitute the volume have stamped their own opinions upon the material gathered. The paper upon *Practical Medicine*, by Dr. Clarke, is worthy of its learned writer. No other man is so well fitted to write a "History of the Discovery of Modern Anæsthesia" as Prof. Bigelow. He has collected all the correspondence which passed between the men, each of whom claimed the honor of discovering the anæsthetic properties of sulphuric ether. Prof. Gross is pre-eminently the man to write the history of surgery. Prof. Thomas and Dr. Billings have contributed valuable and instructive papers. The entire work is one of which the American profession may well be proud. A brief resumé of the book is all that can be attempted in this review. Dr. Clarke begins the century by placing Hunter and Bichât as the landmarks from which all other investigators of their time took their reckonings. He calls these two men the "founders of modern physiology and pathology." Our colonists had little time for engaging in scientific experiments. The development of our agricultural, manufacturing and commercial interests was the first thing to be attended to. But the medical profession were not idle; they found time for some experiments and investigations. Among the prominent men who figured in the early history of our country were Rush, Physick and Chapman, of Philadelphia; Hosack, Watson, Francis and Mott, of New York; the Jacksons, Warrens and Bigelows, of Boston, and others. The first medical lectures were given in Philadelphia by Dr. Cadwallader, just prior to 1751. However, the first regular course of scientific medical lectures was given by Drs. Morgan and Shippen, about the year 1765. Now, about two thousand persons annually graduate from the medical colleges of this country. (About one thousand five hundred too many.) Of the physicians who figured in the early history of this country, Dr. Benjamin Rush stands pre-eminent. To quote Dr. Clarke, he was "an ardent patriot, a friend of Washington, a signer of the Declaration of Independence, and was not only eminent as a physician, but was distinguished as a philsopher and scholar." His treatise on Diseases of the Mind, and his observations on Yellow Fever were both valuable. To Dr. Gerhard, of Philadelphia, belongs the credit of demonstrating the differences between typhus and typhoid fever. Dr. Deveze, of Philadelphia, was the first writer who maintained that yellow fever is non-contagious; and Prof. Alonzo Clark, of New York, was the first to show that the pathological change of the liver in this fever is the result of fatty degeneration. Diseases of the chest have found a most intelligent exponent in Dr. Henry I. Bowditch, of Boston. Thoracentesis has come to be recognized as a tolerably safe, and frequently a nesessary, operation; and its recognition as such, by the profession, is almost entirely due to Dr. Bowditch. Says Dr. Clarke, "the principle of M. Dieulafoy's aspirator is the same as that of Dr. Bowditch's exploring trocar and cannula, with suction pump attached." The study of consumption has occupied much attention in the profession. Among the writers and investigators on the subject Dr. Bowditch holds a high place; also Prof. Austin Flint, of New York. The flexible stethoscope was introduced by Dr. C. W. Pennock, and has since been modified and greatly improved by Dr. Cammann, of New York. Richard Bayley, of New York, as long ago as 1781, pointed out the differences between membranous croup and diphtheria. Dr. John Ware has added valuable contributions to the literature of this subject. Prof. J. C. Dalton, of New York, and Dr. S. Weir Mitchell, each in his respective line of work, Physiology and Neurology, have made a brilliant record; but it is impossible in this sketch to touch upon all the points in this excellent paper. According to the U. S. Dispensatory, chloroform was discovered by Mr. Samuel Guthrie, of Sackett's Harbor, N. Y., in 1831. Almost simultaneously Soubeiran, of France, and Liebig, of Germany, made the same announcement. It is evident, however, that none of the three knew anything of the anæsthetic properties of chloroform. Prof. Bigelow knew, personally, all the experimenters with sulphuric ether, was familiar with all the experiments with the agent, and was present at the first surgical operation under it. His essay goes far towards settling the question as to whom the discoverer was. The three claimants were Drs. Wells, Jackson and Morton. We have not space to give the arguments and statements of Prof. Bigelow at length, but his conclusion is this: "*Nobody has ever doubted* that Jenner was the inventor of vaccination, *and nobody should doubt that Morton was the inventor of modern anæsthesia.*"

Prof. Gross, an Englishman himself, gives great prominence to English surgery, which is only just; but he also gives a fair representation of the surgery of America. See the list of names: Rush, Warren, Physick, Dudley, of Kentucky, Dorsey, author of the first treatise on surgery ever published in this country, Ephraim McDowell, the originator of ovariotomy, Nathan Smith, Davidge, of Maryland, Mütter, Mott, and Daniel Brainard. Of Prof. Brainard, Gross says, "A bold operator, a successful practitioner, and an original thinker, he held, for many years, the leadership of surgery in the Northwestern States of the Union." A further mention of the eminent names in American surgery gives us that of Dixi Crosby, for many years the able professor of surgery in Dartmouth College, Dr. Amos Twitchell, of Keene, N. H., and a host of others. Prof. Gross says, "it is not at all likely that America will ever again produce four surgeons of equal renown with Valentine Mott, John C. Warren, Philip H. Physick, and Benj. W. Dudley." For "it is not at all likely that an equal number of young practitioners will again be placed under equally advantageous circumstances for their development." Among the men who have especially signalized themselves in the ligation of arteries are McGill, of Maryland, Mott, Smythe, of New Orleans, J. Kearney Rodgers, Willard Parker, Prof. McGuire, of Richmond, Va., and others. In the treatment of fractures, Lenox H. Hodge, Gurdon Buck, Nathan R. Smith, and Dr. Hodgen, of St. Louis, are conspicuous. Dr. Wm. W. Reid, of Rochester, N. Y., is the man to whom very great credit is due for advocating the reduction of fractures by manipulation; and his fame ought to be, and is, shared by the present professor of surgery in Rush Medical College, Dr. Moses Gunn. The credit of first using the trephine for the relief of inflammation and abcess of bone is generally ascribed to Brodie: but, according to Gross, it should be given to Dr. Nathan Smith, of New Haven, who performed the operation in the latter half of the last century. Dr. Walter Brashear, of Bardstown, Ky., first led the way in amputations at the hip joint. Prof. Thomas has contributed an able paper, and has covered the field of obstetrics and gynæcology quite thoroughly. Our space admits of only a sketch of his work. Dr. James Lloyd, of Boston is believed to be the first physician who devoted himself exclusively and systematically to the practice of midwifery. Dr. S. V. B. Tennant, of New York, was among the first to lecture upon the subject of obstetrics and diseases of women. Ephraim McDowell, of Danville, Ky., fairly electrified (and it may be said horrified) the medical fraternity by the daring operations for the extirpation of ovarian tumors. This remarkable man was ridiculed and derided by the European journals when he first announced his innovation in surgery. Dr. Nathan Smith, in 1809, performed ovariotomy without the knowledge of its having been done by Dr. McDowell. Ovariotomy was not performed in Germany, England and France until the years 1819, 1836 and 1844, respectively. E. R. Peaslee, Kimball, Dunlap, John L. and W. L. Atlee, Wm. P. Dewees, Meigs, Hodge, Bedford, of New York, who first established the custom of clinics for diseases of women in this country, Marion Sims, whose speculum is known the civilized world over, H. R. Stover, Parvin, of Indiana, Byford and others, are mentioned as among our eminent obstetricians and gynæcologists. Dr. Billings has given us first the quantity of books and periodicals heretofore published by us. The footings are about as follows: American medical books, 2,241: reprints and translations, 2,429; medical journals, 332; volumes of transactions of medical societies, 336. The increase in the amount of medical literature published in the United States has been commensurate with the increase in the population of the country. Concerning the quality of these publications, it is unnecessary to say that the range is very wide. Such treatises as those of Gross, Flint, Wood, Stillé, Dalton and others, will compare favorably with the works of European authors on like subjects. A large portion of Dr. Billings' paper is devoted to biographical sketches of American physicians and surgeons. We are not without a medical lexicographer, whose name reflects honor upon the profession. It is not too much to say that Dr. Robley Dunglison holds the same place in the medical world that Noah Webster occupies in the department of general letters. The essay of Dr. Billings will repay a careful perusal. B. W. G.

THE MEDICAL AND SURGICAL HISTORY OF THE WAR OF THE REBELLION. Part II. Surgical Volume II. Prepared under the direction of *Joseph K. Barnes*, Surgeon General U. S. A. By *George A. Otis*, Assist. Sur. U. S. A.

The present volume of 1024 quarto pages continues a presentation of facts regarding wounds and injuries, according to regional classification, through four chapters, numbered continuously with those of the first surgical volume.

Two hundred and eight pages (chapter VI.,) are devoted to a consideration of contusions and wounds of abdominal parietes, vesical injuries without external wounds, penetrating wounds of the abdomen and abdominal effusions, a tabulation of 8,538 cases of wounds, with a record in detail of 610 cases.

Injuries of the pelvis are considered in chapter VII., which embraces 215 pages. Under this head are included shot fractures of the pelvic bones, injuries of the parts contained in the pelvis, and injuries of the genital organs. Thirty-one hundred cases of wounds of the pelvis are enumerated, six hundred and ten being detailed.

In chapter VIII. some reference is made to flesh wounds of the back.

The concluding chapter upon wounds and injuries of the upper extremities, covering nearly 600 pages, presents a summary of facts pertaining to sword, bayonet and other cuts, stabs and shot wounds. The materials are arranged according to that classification which bases the principal divisions on regional, and the subdivisions on structural characters. The facts reported concerning punctured, incised and shot wounds of the upper extremities are distributed in eight lectures, treating, respectively, of flesh wounds, shot fractures of the clavicle and scapula, wounds of the shoulder joint, shot fractures of the humerus (shaft), wounds of the elbow joint, fractures of the bones of the forearm, wounds of the wrist joint, shot fractures of the metacarpus and phalanges. The work is illustrated in the most creditable manner. We can only, however, note the scope of that volume as a part of a work of which the profession of our country may justly feel proud. J. E. O.

I.—A Treatise on the Science and Practice of Midwifery. By *W. S. Playfair, M.D., F.R.C.P.* Philadelphia: H. C.Lea. 1876. Pp. 576.

II.—A Manual of Midwifery. By *Alfred Meadows, M.D.* Second American, from the third London edition. Philadelphia: Lindsay & Blackiston. 1876. Pp. 490.

III.—A System of Midwifery. By *William Leishman, M.D.* Second American, from the second English edition. Philadelphia: Henry C. Lea. 1876. Pp. 776.

IV.—The Student's Guide to the Practice of Midwifery. By *D. Lloyd Roberts, M.D.* Philadelphia: Lindsay & Blackiston. 1876. Pp. 317.

I. In the preface of this recent contribution to obstetric literature, the author expresses it as his object to place in the hands of his readers an epitome of the science and practice of midwifery, which embodies all recent advances. In a word, we think the writer's object is well accomplished in the treatise.

Probably in no other department of medicine has greater advancement been made than midwifery. To be satisfied with the force of this, one has only to contrast Dr. Playfair's humane views, recommending the frequent use of the forceps, with the inhuman proscription of this great aid of a few years ago; and this is but one of a great many points of contrast. Part V. of the book, in which is treated the puerperal state and its management, contains so much that is new, that the old practitioner must feel that he is reading a new science. This part is of great value to the practitioner.

We cannot recommend this book to the beginner; in fact, it obviously was the author's aim to write a book solely for the advanced student and practitioner. The book is not sufficiently elementary. There is a lack of explicitness in description and definition which precludes this for the first book. It may be argued that the definition of natural labor, for example, is but conventional, and really not important to be remembered in practice. Still, we think it essential that the student should early commit to his understanding certain usual conditions and phenomena, to serve as points of departure. Subsequently, when in practice, it will be easy and perhaps proper for him to look upon the divisions and stages of labor, and of other processes, as arbitrary, and intended for the learner. Besides this objection, the writer is not always accurate; as for instance, on page 93, he says the umbilical arteries are divided from the abdominal aorta. While this is not strictly an anatomical untruth, it is more exact to say, and surely well for the beginner to know, that the umbilical arteries arise from the internal iliacs.

The style of the writer is generally clear and readable. The type and general appearance of the book are attractive. Finally, we should not omit to say that the usual obstetric cuts are generally interspersed throughout the book; the first two pages are embellished with two excellent plates taken from a vertical section of the frozen body during the ninth month of pregnancy.

II. This book has been favorably known in this country for a long time, and deservedly so; for we are not acquainted with a more concise, at the same time complete manual, than this. We especially recommend it to students to read in connection with their lectures on midwifery, when their time is too limited to read a larger treatise. The author still calls his book a manual; in reality it is wholly unlike the multitude of obstetric hand-books, and may well be called an abridged treatise on midwifery. The recent revision and enlargement that the book has undergone have very noticeably increased its value.

That this book is recommended as a text-book by many of the leading scholars of medicine in this country is sufficient evidence of the favor in which it is held. In a word, we know of no better book on the subject in our language, for both the student and practitioner. The value of the book is enhanced by this second edition, which contains many notes by our countryman, the late Dr. Parry, whose work on extra-uterine pregnancy attests his ability to edit a treatise on obstetrics.

IV. This is a small hand-book which the author says "is written mainly for instruction of students." We repeat what we said of another book of this character, that we question the usefulness of hand-books in any department of medicine, especially of obstetric hand-books. They are in every respect too incomplete for the student, and what practitioner would be satisfied with a book of reference that contains so little! We do not doubt that this is quite as valuable as other books of its class. E. W. S.

CLELAND'S DISSECTIONS; a directory for the dissection of the human body. By *John Cleland, M.D., F.R.S.*, Professor of Anatomy and Physiology in Luren's College, Galway. Philadelphia: H. C. Lea. 1877.

This is a neat little volume of 182 pages, of convenient size to carry in the pocket. The subject matter is well arranged, the paper fine, printing clear and distinct. Altogether, it presents a very attractive appearance. It is a matter of no little importance to the student engaged in practical anatomy to know how best to take the body to pieces, so as to show everything to the greatest advantage. The "Directory" will tell him exactly how to do this; so, with its aid, supplementing the admirable work of Gray, dissecting is rendered much more profitable than it would otherwise be.

It does not give the origin and insertion of the muscles, but tells where and how to cut or turn them aside, so as to expose to best advantage the parts beneath.

We cannot too highly recommend it to the zealous student who wishes to make the most of his opportunities while studying anatomy in the dissecting room. A. B. S.

EPITOME OF SKIN DISEASES, WITH FORMULÆ, FOR STUDENTS AND PRACTITIONERS. By *Tilbury Fox, M.D., F.R.C.P.*, Physician to the Department for Skin Diseases in University College Hospital, etc., and *T. C. Fox*, *B.A.*, (*Cantab.*,) *M.R.C.S.* Philadelphia: Henry C. Lea. 1876. Pp. 120.

It is said that for every article which has been offered for sale, sooner or later a purchaser has been found. Even the now traditional coffin with a plate inscribed with the name of Thompson, was finally knocked down to a genuine bidder.

We suppose that the large class of men who deliberately choose to buy epitomes, hand-books and pocket editions will always be with us. We wonder who they are and for what they purchase these wares, but the mystery is never cleared up. They must outnumber all other buyers. The consequence is, that whatever these pigmy publications may or may not contain in the way of information, there is always "money in them." Few medical gentlemen of repute care to provide themselves with these pocket companions, and medical students, as a rule, are too hungry to be satisfied with such light repasts of literature.

For those who desire to find the subject of seborrhœa condensed into a single small page, and of eczema into two equally small pages, this little volume will become a perfect treasure. For him who desires to learn how to combine borax, glycerine and rice water, or calomel and lard, these pages will prove a sapient counsellor. It is true that some of the conspicuous carelessness of the elder Fox, in his large and really valuable treatise is here reproduced. It is true, also, that the use of such books tends to make the student superficial, and the practitioner careless. But what of that? This very little book, written by one great man, assisted by his son, who is not yet great, will be purchased by thousands. It will pass through subsequent editions, will help some men, will hurt others, and will be succeeded in turn by a still smaller volume, written by some author who is clever enough to put the text in smaller type. And there are those who will read this single page on psoriasis, and think they have mastered the subject, when, if their eyes were but opened, they would see the whole field of pathology lying mist-covered before them.

HEALTHY SKIN: A POPULAR TREATISE ON THE SKIN AND HAIR, THEIR PRESERVATION AND MANAGEMENT. By *Erasmus Wilson, F.R.S., F.R.C.S., etc., etc.* Philadelphia: Lindsay & Blakiston. 1876. Pp. 306. 8th edition.

Certainly a desirable end would be gained if every gentleman who had attained eminence in some special department of medicine, could write a good, popular treatise on the subject upon which he is presumed to speak with authority. Such works, if they could secure general circulation, would prove valuable as one means of dispelling the erroneous views of the public upon many medical subjects. But, unfortunately, the successful author in science has rarely the training and instinct requisite for such a task. The success which he has attained in one direction, actually disqualifies him for progress in the other. Lord Macaulay, fond as he was of children, would probably have failed if he had undertaken to write a book especially for his little friends.

Some of the chapters of the popular treatise before us, especially those upon the hygiene of the skin and hair, are admirably adapted to the end in view. But of the work, as a whole, we must express the conviction that it is of the class which may convey that dangerous thing, "a little knowledge," to the ignorant reader. He who is capable of reading it intelligently, and of being sufficiently interested in the subject to read it carefully, had better purchase at once Mr. Wilson's larger work. The non-professional reader who has once wrestled with "molluscum contagiosum," "plica polonica" and "calcareous miliary tubercles" in this *popular* work, surely should be sufficiently advanced in his nomenclature to be able to dispense with such terms as "St. Anthony's fire," "red gown," "humid scall," "mattery pimples," *et id omne genus.*

When patients begin to use their own depilatories, and to treat themselves for "rupia" and "ecthyma," then it would seem to us to be time for the physician to take his leave.

